# Visual Features Assisted Robot Localization in Symmetrical Environment Using Laser SLAM

**DOI:** 10.3390/s21051772

**Published:** 2021-03-04

**Authors:** Gengyu Ge, Yi Zhang, Qin Jiang, Wei Wang

**Affiliations:** 1 School of Computer Science and Technology, Chongqing University of Posts and Telecommunications, Chongqing 400065, China; D190201004@stu.cqupt.edu.cn (G.G.); D180201007@stu.cqupt.edu.cn (Q.J.); D190201021@stu.cqupt.edu.cn (W.W.); 2 Advanced Manufacturing and Automatization Engineering Laboratory, Chongqing University of Posts and Telecommunications, Chongqing 400065, China

**Keywords:** localization, robot, symmetrical environment, laser rangefinder, SLAM, grid map, Monte Carlo localization, visual features

## Abstract

Localization for estimating the position and orientation of a robot in an asymmetrical environment has been solved by using various 2D laser rangefinder simultaneous localization and mapping (SLAM) approaches. Laser-based SLAM generates an occupancy grid map, then the most popular Monte Carlo Localization (MCL) method spreads particles on the map and calculates the position of the robot by a probabilistic algorithm. However, this can be difficult, especially in symmetrical environments, because landmarks or features may not be sufficient to determine the robot’s orientation. Sometimes the position is not unique if a robot does not stay at the geometric center. This paper presents a novel approach to solving the robot localization problem in a symmetrical environment using the visual features-assisted method. Laser range measurements are used to estimate the robot position, while visual features determine its orientation. Firstly, we convert laser range scans raw data into coordinate data and calculate the geometric center. Secondly, we calculate the new distance from the geometric center point to all end points and find the longest distances. Then, we compare those distances, fit lines, extract corner points, and calculate the distance between adjacent corner points to determine whether the environment is symmetrical. Finally, if the environment is symmetrical, visual features based on the ORB keypoint detector and descriptor will be added to the system to determine the orientation of the robot. The experimental results show that our approach can successfully determine the position of the robot in a symmetrical environment, while ordinary MCL and its extension localization method always fail.

## 1. Introduction

There are many positioning methods for mobile robots. A GPS signal is generally used for an outdoor environment [1], while Wi-Fi, ultra-wide band (UWB), radio frequency identification (RFID), and two-dimensional codes are used for indoor environments as artificial beacons where the GPS signal is weak or cannot be received [2,3]. For an autonomous mobile robot to move in an unknown environment without an artificial beacon requires a map. At present, the most popular technology is SLAM in the absence of a prior map: that is, a robot locates itself according to the surrounding environment data collected by sensors, and reconstructs the environment map according to its estimated position and orientation. The commonly used SLAM sensors are laser rangefinders and vision cameras. A 2D laser rangefinder is usually used indoors, while a 3D laser rangefinder is used in an outdoor environment. Cameras are divided into monocular, binocular stereo, fisheye, panoramic camera, RGB-D depth camera, event camera, etc. [4], all of which are suitable for indoor and outdoor environments. With visual SLAM it is difficult to achieve stability and robustness because the images collected by the camera will be affected by an illumination change or textureless area. In addition, a pure visual feature points map is not suitable for flexible path planning and navigation. Due to the distance and measurement information of the laser beam, laser rangefinder sensors are commonly used to realize mapping and positioning in an indoor, structured environment.

From the earliest sonar sensors to the popular laser rangefinder sensors, the SLAM system based on a rangefinder is more reliable and robust. However, due to uncertainty and sensor noise, early processing methods all use probabilistic approaches, which refer to mathematical derivations of the recursive Bayes rule. The invention of an occupancy grid map provides great convenience for rangefinder slam mapping tasks. The rangefinder range measurements provide maps of the robot’s environment, with regions classified as empty, occupied, or unknown, and matches new maps with old ones for landmark classification and to obtain or correct the global positioning and orientation information [5]. The advantage of an occupancy grid map is that it can be flexibly used for path planning. When a map of an environment is completed, a robot comes to the environment again and needs to solve the localization problem, which is a major area of interest within the field of mobile robotics. Global localization, position tracking, and kidnapped robots are the most significant current discussions on localization problems; global localization is most difficult and an increasingly important area in this field. Based on a known map and sensor data of the surroundings, which specify the position of the robot in the environment, almost all robot localization problems in asymmetrical environment can be solved by the Monte Carlo Localization algorithm [6] and its extended algorithms [7,8].

**Figure 1:**
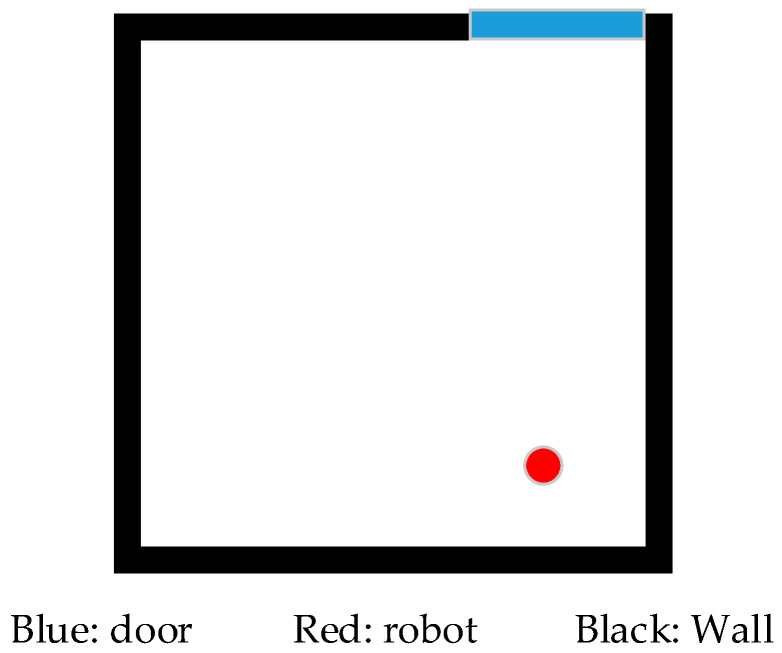
A square, symmetrical room.

Symmetrical environments are common in the manmade, structured world, especially indoor scenes, where building and room layouts are symmetrical. Most of them are symmetrical before placing furniture or other sundries. Viewing from the perspective of 2D lidar at a certain horizontal altitude, many common regular polygon environments in indoor scenes can be regarded as circular, square, or rectangular symmetric environments. When a robot comes to a square symmetrical indoor space with the door closed, as shown in Figure 1, the single laser scanning beam distance data and location method based on a probability map cannot uniquely determine the position and orientation of the robot when its initial position is unknown. Obviously, the position of the robot can be uniquely determined at the geometric center of the square, but the orientation cannot be determined, because there are four possible orientations related to the number of sides of a regular polygon. In addition to the position of the geometric center, the robot has four possible positions because the surroundings seen by the robot are the same. If the robot does not go out of the door, it cannot refer to the features outside the door for localization. The existing approaches based on probabilistic location algorithms will be invalid.

In this paper, we propose a visual features-assisted approach to solve the above problems. An additional monocular camera will be mounted on the robot to extract image features in the vertical direction in front of the robot and determine the robot’s orientation according to the differences between four directional images. The main contributions of this article are as follows:

- This paper proposed an approach to judge whether an environment is symmetrical or not. Using the exclusion method to eliminate the asymmetry cases, we then extracted corners and fitting lines to test the hypothesis. The experimental results showed that this approach is effective, especially for a square environment, as in the case in this paper.
- This paper proposed a visual features-assisted method to help a robot localize in a symmetrical environment. ORB features and the bag-of-words method are used to describe an image, which are robust and efficient for image matching.
- We designed an algorithm to make the robot move to the geometric center position and capture several discrete images, which are used to decide the orientation. Compared with other popular methods, our results show that less memory and disk storage space are needed.

The rest of the paper is organized as follows. Section 2 gives an overview of related work. Section 3 provides an overview of the map-building and localization process. Section 4 presents the main methods including laser data preprocessing, judgement of symmetrical environment, visual features extraction and representation, and localization. In Section 5, we test our methods in both simulation and real-world environments, and analyze the results. Section 6 discusses the paper and draws conclusions.

## 2. Related Work

### 2.1. Laser-Based SLAM

SLAM can solve a localization problem of a robot in an unknown environment without a prior map. The earliest concept was put forward by Smith and Cheeseman [9] in 1986. The SLAM problem can be formulated in two ways. The first is to estimate the current position of a robot, usually based on the latest sensor information, known as online SLAM. The second is to estimate the whole trajectory of a robot and map according to all the control inputs, odometer, and measurements, which is called full SLAM [10]. Filter-based methods derive from Bayesian filtering and work as prediction and updating (a two-step iterative process); they are suitable for solving the problem of online SLAM [11]. EKF SLAM based on the Extended Kalman Filter was first used in sonar sensors for underwater applications, then gradually used in laser and vision sensors [12]. Another major branch in filtering SLAM algorithms is based on particle filters, which do not require a Gaussian noise assumption and can accommodate any distribution. Robot position state is sampled with a set of particles according to its probability density, and particles are weighted according to their likelihood regarding the measures in the update phase. The most famous work is Gmapping [13], which is based on the Rao-Blackwellized particle filter algorithm and is integrated into the Robot Operating System (ROS) used by many commercial robots. Graph-based approaches use a matrix describing the relationships between landmarks and robot positions can be built easily and used in an optimization framework, which can allow one to find the minimum of a cost function. The classic solution based on graph SLAM is Cartographer [14], which was invented by a team of engineers at Google and also integrated into ROS. Generally, both Filter-based SLAM approaches and Graph-based algorithms have developed accuracy in map construction in indoor, small-scene environments. However, for a large-scale environment such as a factory or workshop, it is recommended to use the scheme of graph optimization.

### 2.2. Feature Extraction

Among the different types of sensors, 2D laser range finders provide accurate range distance measurements, high sampling rates, and angular resolution, and have become increasingly popular in mobile robotics. However, raw sensor data without artificial landmarks cannot be used to estimate the position of a mobile robot or to build a map of an unknown environment. Geometric features such as corners, breakpoints, endpoints, lines, curves, etc. are always extracted to help locate or map tasks. Line segments are the most basic and simplest among all geometric features extracted from 2D laser range data. The corner point, which is an important landmark, can be found from the intersection of two adjacent line segments [15]. Line extraction algorithms using 2D range data for indoor mobile robotics are compared in [16], including Split-and-Merge, Incremental, Hough-Transform, Line-Regression, RANSAC, and the EM algorithm. The comparative experiments conclude that the Split-and-Merge and Incremental methods provide the best performance among the existing algorithms, while the former is clearly the best choice for its superior speed. Another line segment extraction based on the seeded region growing approach shows better results than Split-and-Merge [17], through the design of seed-segment detection, region growing, overlap region processing, and endpoint generation.

### 2.3. Visual Features

Visual features extracted from images are usually used for place recognition, target recognition, image classification, video description, and object detection. In robotics, a sequence of images is usually used as a visual map for a robot visual navigation task. The survey by Williams et al. [18] shows that an appearance-based image-to-image matching method works better in large environments, while an image-to-map method is the opposite. Another survey article by Lowry et al. [19] lists many visual feature detector algorithms, including local feature descriptors like SIFT [20] and SURF [21], and global or whole-image descriptors such as Gist [22]. The SIFT keypoint detector and descriptor is very stable as it is impervious to rotation, scale scaling, and brightness changes. Many other algorithms use its ideas for reference. SURF (speeded-up robust features), using a Hessian matrix-based measure for the detector and a distribution-based descriptor, can be computed and compared much faster. Later, a novel feature built on the well-known FAST keypoint detector [23] and the recently developed BRIEF descriptor [24], called ORB [25], is two orders of magnitude faster than SIFT. The ORB feature is used in many famous works, such as the ORB-SLAM series, for image matching and loop closure [26,27,28].

Several works similar to ours are [29,30,31], in that their research focuses on the localization problem in a rectangular, symmetrical environment such as the RoboCup soccer field. In [29], an approach called Uniform Clustered Particle Filtering MCL is based on Uniform MCL and Clustered Particle Filtering MCL algorithms. When executed in a symmetrical environment, after iterating several steps, particles accumulate in several locations. The number of locations is determined by the number of symmetrical shapes, so cannot be uniquely determined. The authors of [30] used a simple clustering algorithm to separate the particles into different clusters based on the Monte Carlo localization framework. However, the system needs a known orientation and the robot’s initial orientation. This is to determine which cluster corresponds to the direction of the robot. Brindza et al. [31] propose a solution to the symmetrical field problem that involves the use of sound emission and detection to determine the direction of the defending and attacking goals relative to a robot’s current position, which is used in the RoboCup competition.

## 3. System Overview

**Figure 2:**
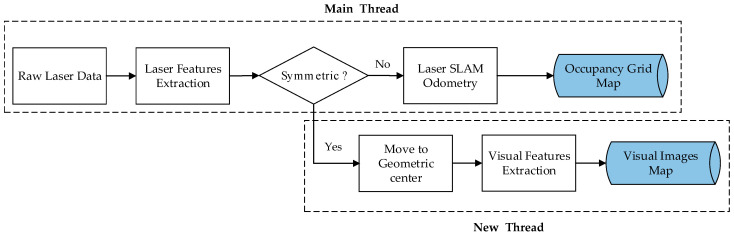
Map-building process.

**Figure 3:**
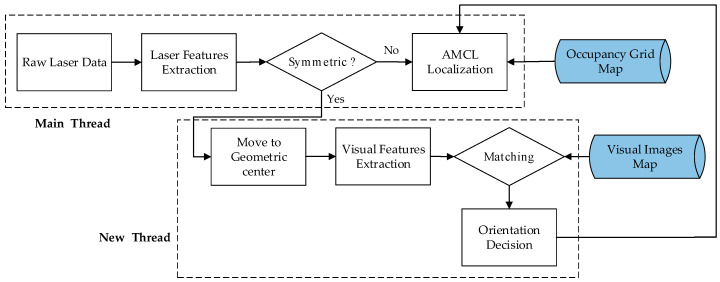
Localization process.

The proposed visual features-assisted robot localization framework is divided into two stages, which are shown in Figure 2 (the map-building process) and Figure 3 (the localization process).

In the map-building stage, a robot cannot locate its position when it comes to an unknown environment. A mapping system using laser-based SLAM framework to build an occupancy grid map needs both raw laser scan data from an external sensor and odometer information from an encoder (internal sensor acquisition). Compared with traditional SLAM systems, this work adds a module of symmetrical 2D space judgement, and removes the asymmetry cases by using the exclusion method. Then, a line extraction algorithm is used to fit a straight line and extract corner points from intersection adjacent lines. If the Euclidean distances between adjacent corners are all equal, then the two-dimensional geometric feature of the surrounding environment is likely to be a square, symmetrical environment. The robot should move to the geometric center of the symmetrical environment and small errors will be ignored; it captures images from the direction facing the middle of two adjacent corners. ORB descriptors will be extracted from these images, then calculated and converted to visual words based on DBoW3 [32], which is an improved version of the DBow2 library [33], an open-source C++ library for indexing and converting images into a bag-of-words representation. The visual words and images will be saved in the visual images map database.

When a robot comes to a place it passed before in the localization stage, the previous built occupancy grid map can be used for localization. The robot can locate itself in an asymmetrical environment by using the Adaptive Monte Carlo Localization (AMCL) algorithm [34], which is integrated into the ROS navigation tool kit [35]. In the process of the robot moving forward, laser data are extracted to judge whether it is a symmetrical environment. If it is a symmetrical environment, the robot moves to the geometric center, then extracts visual features, generates visual words, and matches them with the visual images map database built before. After the matching is successful, the orientation of the robot can be determined. At this time, the AMCL algorithm can work normally.

## 4. Methods

### 4.1. Laser Data Preprocessing

**Figure 4:**
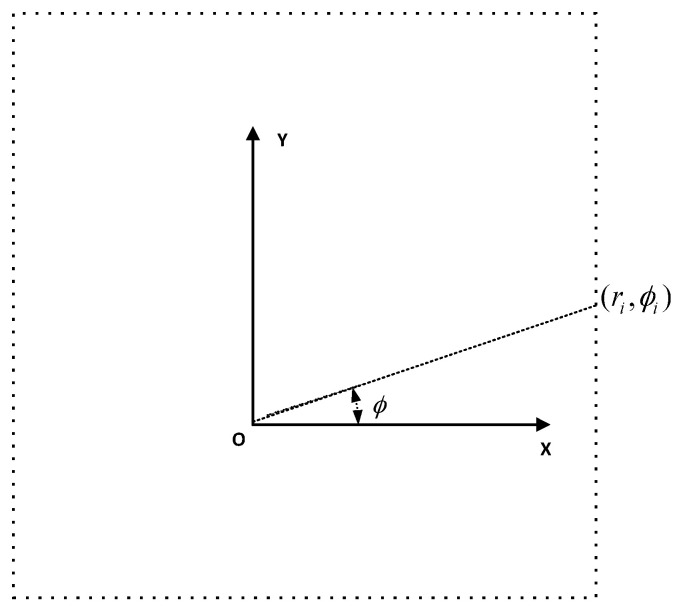
Laser ranger scans from a square structure environment.

Most commercial 2D laser rangefinders are based on a fixed time-of-flight laser measurement system, which can only measure distances in a given direction of 360 degrees around the center of the laser sensor. The basic parameters of a laser rangefinder include the maximum measurement distance $D_{\max}$, minimum measuring distance $D_{\min}$, minimum angle interval or angular resolution $\phi_{\min}$, total number of scanning points $N=360/\phi_{\min}$, and the ith scan data $\left(r_{i},\phi_{i}\right)$, in which $r_{i}$ means the distance between the *i*th laser beam from the emission point to the obstacle, while $\phi_{i}$ means the angle between the ith laser beam and the positive x-axis and the counter clockwise direction is taken as the positive direction. The raw data form is $R=\{\left(r_{i},\phi_{i}\right)|i=1,\ldots ,N\}$, as shown in Figure 4 from a square environment similar to Figure 1; the center of the laser sensor is the origin of the polar coordinate system. Convert raw data $P=\{\left(x_{i},y_{i}\right)|i=1,\ldots ,N\}$ and ignore the angle parameter.

(1) $$x_{i}=r_{i}\cdot \cos\phi_{i}$$

(2) $$y_{i}=r_{i}\cdot \sin\phi_{i}$$

The traditional method is to fit all points into multiple lines, extract the intersection points of adjacent line segments as corner points [36], and then calculate the distance between adjacent corners. If these distances are equal, it is a symmetrical environment. Although most of the artificial buildings in the real world are symmetrical, a variety of objects used by human beings are placed inside the space, resulting in an asymmetrical environment. Most of the indoor local areas will appear to include a lot of scattered lines. If we use a traditional method, this will waste a lot of computing time.

### 4.2. Judgment of Symmetrical Environment

**Figure 5:**
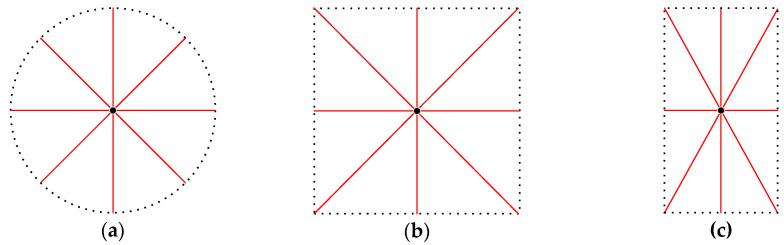
Common regular polygon environments in indoor scenes. (**a**) Circular area; (**b**) square symmetric environment; (**c**) rectangular symmetric environment.

This section takes a square structure environment as an example to describe the algorithm that discriminates whether the environment is symmetrical. Two concepts need to be explained first: the geometric center is generally for a regular polygon, while a geometric centroid is the average of all vertex coordinate values of a polygon with the same weight value of 1. If a convex polygon is centrosymmetric, the two points are the same. A centrosymmetric convex polygon has distinct characteristics such as the distance from the center point to each vertex being equal, like in the three cases shown in Figure 5, where the distance can be thought of as the longest distance from the center point to the edge. These three cases are the most common closed and symmetrical indoor environments. When it is a regular polygon like Figure 5b, all angles are equal and the distance from the center point to each middle of an edge is equal, which can be thought of as the shortest distance from the center point to the edge. Moreover, each edge of a polygon has the same length. If in a circular area like Figure 5a, all radii of a circle are equal, it can be thought of as a regular N-polygon, where N has a value of infinity.

The geometric centroid $P_{centroid}=\left(x_{centroid},y_{centroid}\right)$ is defined as follows:

(3) $$x_{centroid}=\frac{1}{N}\overset{N}{\underset{1}{\sum }}x_{i}$$

(4) $$y_{centroid}=\frac{1}{N}\overset{N}{\underset{1}{\sum }}y_{i}.$$

The new Euclidean distance between the contact point of the laser emission line to the obstacle and the geometric centroid $R^{\prime }=\{r_{i}{}^{\prime }|i=1,\ldots N\}$ is defined as follows:

(5) $$r^{\prime }=\sqrt{{\left(x_{i}-x_{centroid}\right)}^{2}+(y_{i}-y_{centroid}{)}^{2}}.$$

Based on the above facts, in order to improve the speed of calculation, which determines whether the environment is square symmetrical or not, the method shown in Algorithm 1 is proposed.
**Algorithm 1.** Judgement Method of Square Symmetrical Environment**Input:** A set of N points $R=\{\left(r_{i},\phi_{i}\right)|i=1,\ldots ,N\}$**Output:** Symmetrical environment or not1: Convert raw data R to coordinate data $P=\{\left(x_{i},y_{i}\right)|i=1,\ldots ,N\}$, ignore angle parameter2: Calculate geometric centroid $P_{centroid}$
3: Calculate new distance data $R^{\prime }$ from P
4: Descending sort $R^{\prime }$ as $R^{\prime \prime }$
5: Count the number of maximum values $num_{\max}$ and number of minimum values $num_{\min}$
6: If $\left(num_{\max}\neq 4||num_{\min}\neq 4\right)$ then7: Environment is not square symmetrical8: End if, terminate9: Calculate the Euclidean distance between two adjacent points $d_{12}$, $d_{23}$, $d_{34}$, $d_{41}$ in turn 10: If $d_{12}$, $d_{23}$, $d_{34}$, $d_{41}$ are not equal then11: Environment is not square symmetrical12: End if, terminate13: Fit 4 lines by connecting adjacent two points in turn from 4 maximum distance points14: Count the vertical distances from the remaining points to the 4 lines15: If all distances less than ε which infinitely close to zero then16: Environment is square symmetrical17: Save the G‒V distance to the G‒V index list18: else19: Environment is not square symmetrical20: End if, terminate

In Step 5 of Algorithm 1, these equal maximum values can be assumed to be the distance from the center point to each vertex, while the equal minimum values are the distance from the center point to each middle of an edge. In Steps 6 to 8, if $num_{\max}$ equals $num_{\min}$ and both of them equal N, then the scene is very likely to be a circular area.

In Step 17 of Algorithm 1, the G‒V distance is defined as the geometry centroid or center point to one of the vertex points in a symmetrical environment. A G‒V index list will be created to store the G‒V distances of different symmetrical environments so that the number of image matching times can be reduced.

### 4.3. Visual Features’ Extraction and Representation

In a closed symmetrical environment, the distances of laser scans can determine some scale and metric information, and the orientation of a robot can be determined by visual features information. The first thing the robot needs to do is move to the geometric center position, which is the geometric centroid position when the environment is a centrosymmetric regular polygon like a square. The specific implementation method is shown in Algorithm 2.
**Algorithm 2.** Robot Moves to Geometry Center Position**Input:** Coordinates of Geometric Centroid**Output:** Robot Moves to the Proper Position1: If environment is square symmetrical, then2: Let geometric center coordinates equal to geometric centroid coordinates 3: Robot moves towards the geometry center position4: Calculate the middle of two adjacent corner points 5: Robot adjusts its orientation to the middle of the two adjacent corner points6: else if7: Terminate8: End if

Most place recognition methods using visual features extracted from images need to consider the robot or camera position information, which includes position and orientation. The most commonly used scheme is based on the bag-of-words model, which is used in many place recognition and loop closure detection systems [37]. The idea of the bag-of-words approach in image retrieval comes from a text retrieval system [38], which is the first systematic application to solve the problem of object retrieval in videos. The image features process is divided into offline and online stages. In the offline stage, a visual vocabulary is created from a large number of images from the general environment by discretizing the descriptors space into W visual words. In the online stage, a new image captured by a robot will be converted into a bag-of-words vector $V_{i}$. Considering image features such as rotation invariance, scale awareness, time consumption, and resistance to noise, the ORB feature is the most suitable to be extracted. The whole process is shown in Algorithm 3.
**Algorithm 3.** Visual Features Extraction**Input:** M images from M directions in Node i, M equals to 4 in a square environment**Output:** Bag-of-words vector $V_{ij}$, where j is the number of image in Node i1: Create a visual vocabulary in an offline step based on DBoW3 with ORB features 2: Robot captures images from four directions, $I_{i1}$, $I_{i2}$, $I_{i3}$, $I_{i4}$
3: Extract ORB features from each image4: Covert features of each image into a bag-of-words vector, $V_{i1}$, $V_{i2}$, $V_{i3}$, $V_{i4}$
5: Save the vectors to an image map database

The input in Algorithm 3 should indicate where the images were collected by using a topological node number of the symmetrical environment. The value of i is usually not too large because there are a few closed symmetrical environments in real-world or manmade surroundings. In Step 5, the four directions cannot be used to distinguish the main direction, so these vectors can be stored in clockwise order to avoid the disorder of subsequent comparison.

### 4.4. Localization

When a domestic robot moves in an indoor asymmetrical environment, the occupancy grid map obtained from laser scans can be used for localization reference. The most popular location algorithm is MCL and its extended versions. The basic MCL algorithm estimates the robot position using a particles filter method in which the set of M particles $\chi_{t}=\left\{x_{t}^{\left[1\right]},x_{t}^{\left[2\right]},\ldots ,x_{t}^{\left[M\right]}\right\}$ represents the belief confidence $bel\left(x_{t}\right)$ at time t. The first step is to randomly generate a bunch of particles, the second step is to predict the next state of the particles, the third is to update the weighting of the particles based on the latest measurement, then resample, while the last step is to compute the estimation. The inputs of the algorithm are $\chi_{t-1}$, which is the particle state of the last moment t−1, current moment motion state $u_{t}$, current moment measurement state $z_{t}$, and prior map m. The main steps of MCL are shown in Algorithm 4.
**Algorithm 4.** MCL Algorithm**Input:**$\chi_{t-1},u_{t},z_{t},m$**Output:**$\chi_{t}$1: Initialization, ${\overset{‒}{\chi }}_{t}=\chi_{t}=Ø$
2: For j=1 to M do3: $x_{t}^{\left[j\right]}=$ sample_motion_model $\left(u_{t},x_{t-1}^{\left[j\right]}\right)$
4: $w_{t}^{\left[j\right]}=$ measurement_model $\left(z_{t},x_{t}^{\left[j\right]},m\right)$
5: ${\overset{‒}{\chi }}_{t}={\overset{‒}{\chi }}_{t}+<x_{t}^{\left[m\right]},w_{t}^{\left[m\right]}>$
6: End for7: For j=1 to M do8: Draw i with probability $\propto w_{t}^{\left[i\right]}$
9: Add $x_{t}^{\left[i\right]}$ to $\chi_{t}$
10: End for11: Return $\chi_{t}$


In Step 3, each particle is a position hypothesis with the motion model:

(6) $$x_{t}^{\left[j\right]}\sim p(x_{t}|x_{t-1},u_{t}).$$

In Step 4, correction is via the observation model:

(7) $$w_{t}^{\left[j\right]}=p(z_{t}|x_{t}^{\left[m\right]}).$$

From Step 7 to Step 10, the weight update rule is

(8) $$w_{t}^{\left[i\right]}=p(z_{t}|x_{t}^{\left[m\right]})w_{t-1}^{i}.$$

The Monte Carlo localization method can solve the global localization problem, but it cannot recover from robot kidnapping or global localization failure. Some improved versions have been proposed, such as Augmented MCL and KLD Sampling MCL, both of which are considered Adaptive Monte Carlo localization methods (i.e., AMCL, which has been integrated into ROS and used by commercial robots). The main problem with using the AMCL method in a symmetrical environment is that no sufficient information can be used to determine robot orientation. Some improved approaches like [29,30] can only localize up to symmetry, that is, more than one localization result was obtained. To solve the problem, a novel approach based on image features is proposed in Algorithm 5.
**Algorithm 5.** Localization Method**Input:** Occupancy Grid Map, Visual Images Map **Output:** Localization Result1: Load Occupancy Grid Map2: AMCL localization method is used by default3: Receive laser scans data and call Algorithm 14: If the environment is symmetrical, then5: Call Algorithms 2 and 36: Compare G‒V distance with G‒V Index List7: Compare new bag-of-words vectors with visual images map database8: If match succeeded, then9: Go to Step 210: else11: Create new visual images map12: End if13: else14: Go to Step 215: End if

## 5. Experiments and Results Analysis

The space surrounded by walls in manmade structured buildings cannot be changed arbitrarily; in addition, too many desks and other furniture are piled up in the authors’ laboratory. Therefore, we did experiments separately in a simulation environment and an indoor testing environment based on the real world. In the simulation environment, parameters of robot and space sizes can be configured arbitrarily; we did experiments to test the algorithm for judgement of symmetrical environments and to localize a robot with only laser scans data from an external sensor. On the other hand, visual features-assisted robot localization experiments were done in a manmade symmetrical testing environment, which was an area enclosed by a rigid plastic board in the laboratory.

### 5.1. Gazebo Simulation Environment

In this part, the hardware platform was a desktop computer configured with Intel(R) Core(TM) i5-7500 CPU at 3.40GHz, 8GB RAM memory, and 500 GB SSD hard disk. Then, we installed ROS Kinetic Kame and Gazebo simulation software based on Ubuntu16.04 distribution Linux OS. The robots were simulated by Gazebo modeling software called turtlebot3, which has three versions, burger, waffle, and waffle pi.

**Figure 6:**
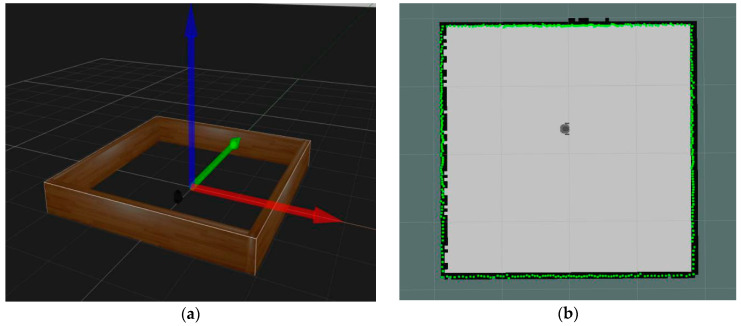
Turtlebot3_burger in a square environment. (**a**) Simulation environment includes turtlebot3_burger and turtlebot3_square model, red arrow as X axis, green arrow as Y axis, and blue arrow as Z axis. (**b**) Discrete points collected by rangefinder sensor from center point to square walls.

First, we modeled a virtual robot named turtlebot3_burger using executed instruction: roslaunch turtlebot3_gazebo turtlebot3_empty_world.launch. The robot was configured with IMU that was used to measure the rotation and acceleration information, and lds_lfcd_sensor as a laser rangefinder was used to measure distances between the robot and the surrounding walls. Then, we inserted a turtlebot3_square model as a square polygon environment that surrounded the robot, as shown in Figure 6.

We did experiments in different centrosymmetric simulation environments, which included an equilateral triangle, square, regular pentagon, regular hexagon, and round wall areas, all of them combined by wall models. Each polygon environment had three different G‒V distance versions and the scanning radius of the rangefinder sensor was configured as 15 m. The robot with rangefinder sensor was evenly placed in 50 different positions in the polygon environment every time and 50 scanning data points were collected to extract the features. Then, the features were used to determine whether the environment was symmetrical using the corresponding algorithm described in Section 4. The experimental results are shown in Table 1, which indicates that this model and algorithm are efficient and suitable to judge a symmetrical environment.

**Figure 7:**
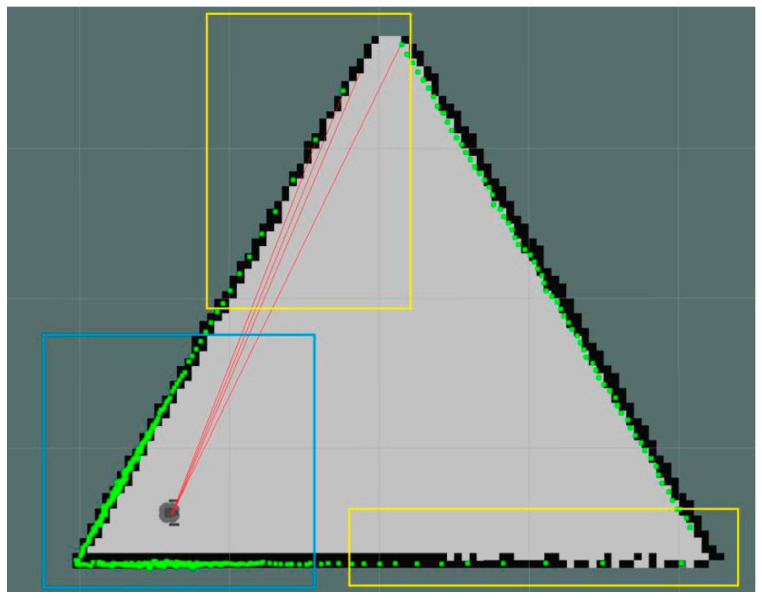
Turtlebot3_burger scanning data in an equilateral triangle environment.

In the last column of Table 1 where the G‒V distance equals 7 m, the algorithm failed several times. Through observation and analysis of data, we found an interesting phenomenon. When the G‒V distance was close to the scanning radius of the laser rangefinder and the robot was close to one of the walls, it was easy to make a wrong judgment. As the scanning interval angle of rangefinder sensor was equal, scanning points from the obstacle wall near the rangefinder sensor were very dense, while scanning points from the other side, far from the robot position, were sparse. The distance between sparse points was relatively long, and it was easy to miss the vertexes of the polygon. A further explanation is shown in Figure 7.

In Figure 7, within the blue rectangle area were dense points because of being close to the robot with a laser rangefinder sensor. Points in the two yellow rectangular areas were sparse. Red lines represent the adjacent scanning distances. Compared with points closer to the robot, the distance between two adjacent points far from the robot was long even if the adjacent angle was equal. The vertex in the top yellow rectangular area had not been detected by the robot because of the above reasons; this led to a wrong judgement on whether an environment was symmetrical or not. Therefore, when the robot moved in the real environment, it needed to avoid moving close to the wall.

**Figure 8:**
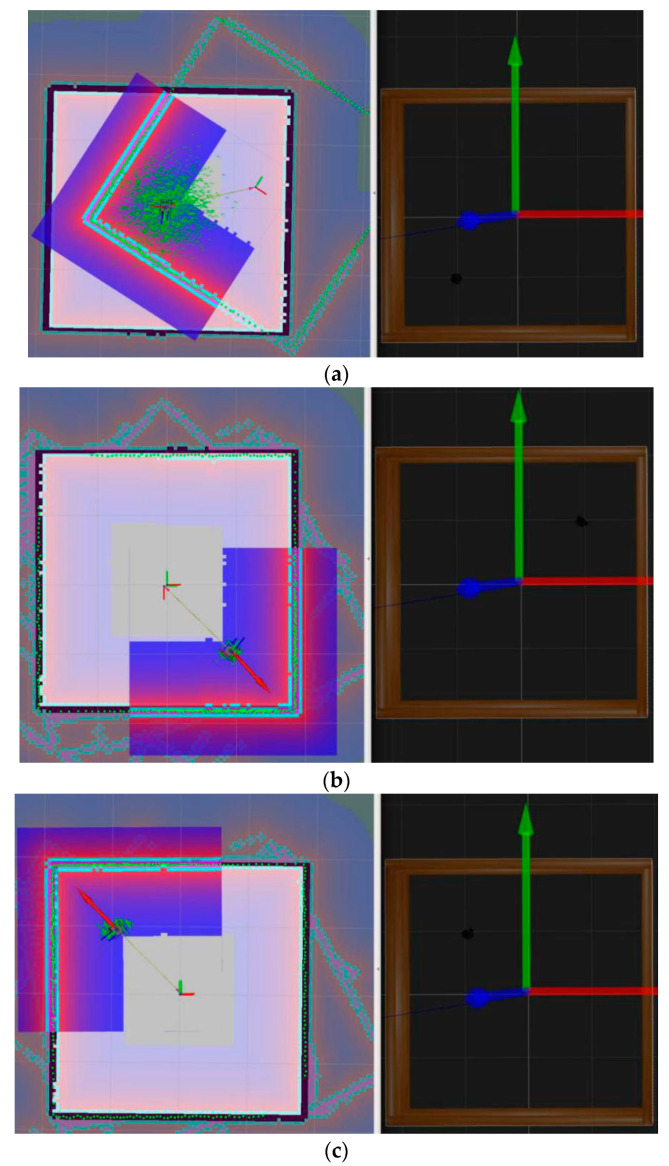
Turtlebot3_burger relocalization and navigation in a square environment. (**a**) Robot initialization with a previously built map. (**b**) Robot relocalization with a manually set wrong position. (**c**) Robot relocalization with a manually set correct position.

A further experiment on robot localization without the visual features-assisted method was done using AMCL localization in a square simulation environment, as shown in Figure 8. At the mapping stage, we chose a Rao-Blackwellized particle filter-based SLAM algorithm, known as Gmapping, which was integrated into ROS. The Gmapping SLAM process combined the data of the robot odometer and external laser scanning distances. With instruction, roslaunch turtlebot3_slam turtlebot3_slam.launch slam_methods:=gmapping, we obtained a probabilistic occupancy grid map and saved it in the system. At the localization or navigation stage, we executed the instruction roslaunch turtlebot3_navigation turtlebot3_navigation.launch map_file:=$HOME/map. yaml. The results showed that the robot cannot localize itself correctly without human assistance.

In Figure 8a, the turtlebot3_burger robot started from a prebuilt occupancy map from the right part, which was a square environment. We found that the newly built square map with pure green contour points did not match the prebuilt map with black contour lines. Moreover, the discrete distribution of particles, which were green points around the robot, indicated the uncertainty of the robot’s position. Therefore, turtlebot3_burger could be in any position near the four corners of the square map.

In Figure 8b, the robot’s real position was near the upper right corner inside the square wall in the Gazebo environment, as shown in the right side of the figure. When we set the robot position to a similar place in the lower right corner, we found that the robot’s newly built map was coincided with the prebuilt map, as shown in the left part figure, along with the two coordinate systems. The turtlebot3_burger robot did not realize its wrong position compared with the right part of the figure, which had the real position, because the features perceived by the robot were almost the same. Further experiments in navigation from the estimation position to the destination position still could not determine what was wrong.

In Figure 8c, the robot was set to the correct orientation and position near the upper left corner of the newly built map, the same as the real position shown in the right side of the figure. With human assistance, this initialed localization method made the particles quickly converge to a concentrated region and obtained the correct position. Then, combined with the robot odometer and laser data, robot localization and navigation experiments consistently maintained the correct trajectory.

The above simulation experiments show that a robot in a symmetrical environment can perceive the polygon using our method and can consistently localize itself with a correct initial position. Orientation can be achieved using human assistance or another method like visual features.

**Figure 9:**
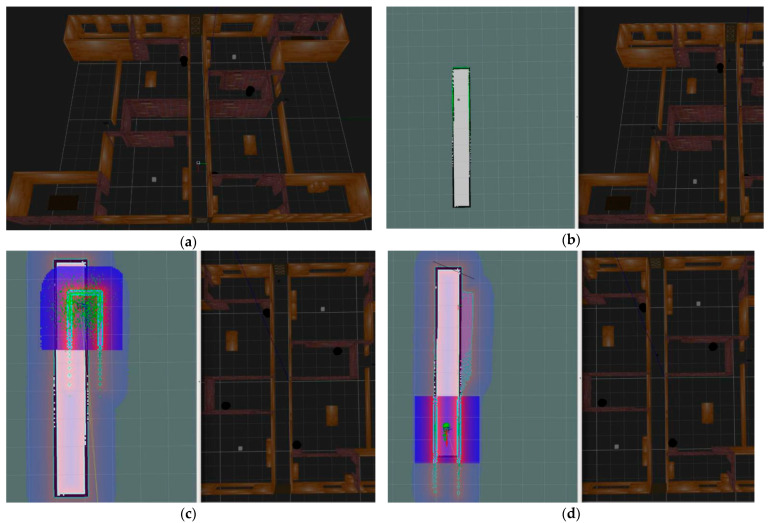
Turtlebot3_burger relocalization and navigation in a narrow hallway. (**a**) Simulation environment includes turtlebot3_burger and a narrow hallway. (**b**) Map of the hallway. (**c**) Robot initialization in the hallway map. (**d**) Navigation in the hallway map.

In fact, a long and narrow corridor or hallway environment is also symmetrical. We did a test experiment in a simulated hallway environment which can be thought as a rectangle, as shown in Figure 9. In Figure 9a, we used two turtlebot3_house models to form a rectangular area as the hallway. In Figure 9b, the robot traversed the hallway and built a map. We found that the length of the constructed map is far less than that of the actual hallway. This is because the diameter of the scanning range of the laser sensor is smaller than the length of hallway, and the odometer has accumulated error. For the same reason, the robot initialization in the hallway map can not relocate correctly in Figure 9c. When we set the robot initial position to the other side of the hallway, the robot still can not realize its wrong position, which is similar as in a square environment.

Unfortunately, when we set a target position close to the opposite side of the hallway, the navigation result is still wrong as shown in Figure 9d. The robot has reached the end of the built map, but actually reached half the distance in the real hallway environment.

From the experiment in corridor or hallway environment, we find that if the diameter of the scanning range of the laser sensor is longer than the length of hallway, human assisted positioning is effective; otherwise the robot can not get correct localization. This aspect of research is involved in [39].

### 5.2. Real-World Environment

In order to further verify the effectiveness and performance of our proposed algorithms, we built an assembled robot platform based on the existing equipment in our laboratory and a square area using a rigid plastic board. Experiments tested the visual features-assisted robot localization.

#### 5.2.1. Robot Platform and Environment

**Figure 10:**
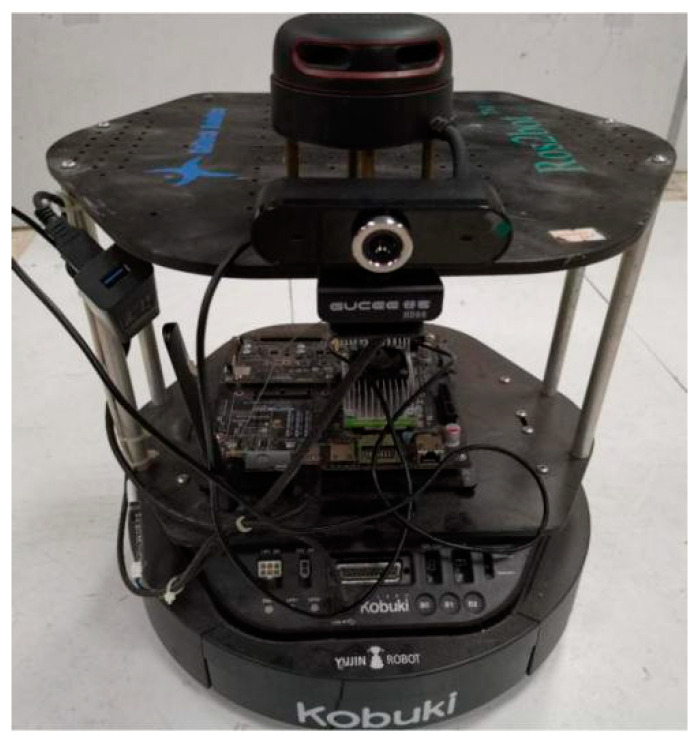
Robot platform and related devices.

We built a robot platform modified from a Kobuki robot chassis (Yujin Robot Company, Seoul, Korea), which only used its mobile chassis, as shown in Figure 10. The mobile chassis has two driving wheels and one driven universal wheel and is suitable for a classical kinematic model. Furthermore, odometer and IMU sensors are integrated in the robot. The on-board computer is NVIDIA Jetson TX2, which has NVIDIA Pascal GPU for large image processing operations or the SLAM algorithm. The laser rangefinder sensor is RPLIDAR A2 (SLAMTEC, China), which has a 16-m measuring radius, a 360-degree scanning range, a 10 Hz working frequency, and 8000 measurements per s. The sensor can satisfy most indoor scenes and scan all surrounding buildings at once. To avoid blocking the scanning, a calibrated monocular camera with 1280 × 960 resolution and 30 frames/s is fixed on the robot platform lower than the laser rangefinder sensor. As the camera is just to achieve an orientation for the robot, the laser-camera calibration work can be omitted. The only thing we need to do is to keep the positive direction of the camera in line with the positive direction of the laser rangefinder.

We installed ROS Kinetic Kame in a Ubuntu16.04 Linux OS, which was installed in the TX2 processor hardware system. Then, a Gmapping SLAM software package that was used to mapping the real-world environment was downloaded and installed in ROS. In addition, a navigation package based on the AMCL localization algorithm, A* global path planning algorithm, and DWA local obstacle avoidance algorithm, was added to the system. Finally, we implemented the laser data processing, visual words converting based on DBoW3, and robot adaptive moving algorithms, using C++ and Python programming language. All implementations followed ROS programming and message communication specification.

**Figure 11:**
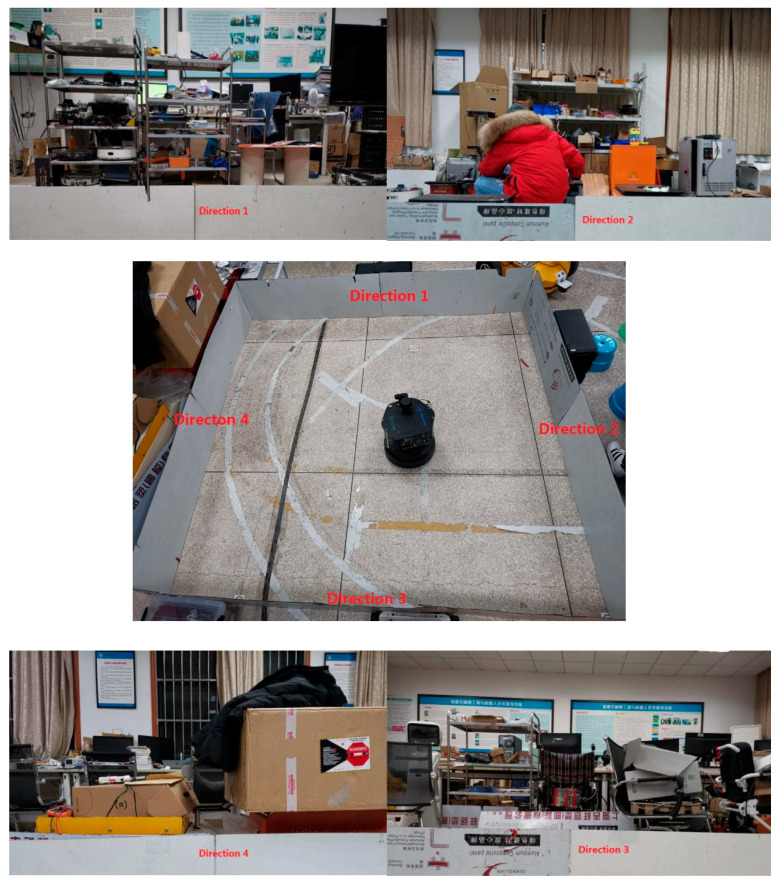
Robot in a real-world environment. The top two and bottom two images are captured from direction 1 to direction 4 and the center image shows the robot in the center of the built square area.

The real-world symmetrical environment we built in the laboratory is shown in Figure 11. The laboratory contains office desks and chairs, experimental equipment, shelves, cartons, and other objects. Moreover, there is a door, several windows, and billboards on the wall. Therefore, the visual texture information of the whole indoor environment is abundant. In the center of the lab, we built a 3 × 3 m square area that was surrounded by several plastic boards of the same size. The central of Figure 11 shows a real robot inside the square environment and four directions are marked toward the middle point of the four sides of the square, in clockwise order. The remaining four parts of Figure 11 are images captured from the above four directions, respectively.

We designed the following experiments to evaluate the effectiveness:

(1) Symmetrical environment judgement and robot movement to the geometry center. The robot was placed in different locations inside the square area and we performed the experiments more than 50 times to test the performance. Each time, the robot started up the software system from initial state, processe laser scans data for symmetrical square judgement, and moved to the geometry center location to capture images in different directions.

(2) Visual features extraction and matching for orientation localization. The experiment was divided into an offline phase and an online phase. First, we collected 1000 images from indoor and outdoor environments near the laboratory, extracted the ORB features, and trained a vocabulary dictionary in the offline phase. Then, at the geometric center of the square and toward the midpoint of each edge, we captured four images in clockwise order. Finally, the ORB features were extracted and converted into a sparse numerical vector from each image. These results were saved as a visual map stored in a database. All of the above work was completed in the offline phase.

In the online phase, the robot was placed in the built square environment, moved to the center position, and randomly selected a direction toward the midpoint of the square edge. Then, it captured an image, extracted the ORB features, and converted them into a sparse numerical vector. Finally, we compared the vector with the previous visual map to decide the robot’s orientation. We repeated the above work three more times in clockwise order if the first pair did not match successfully.

#### 5.2.2. Experimental Results Analysis

(1) Symmetrical Judgment in Different Locations and Deviation from the Geometric Center

**Figure 12:**
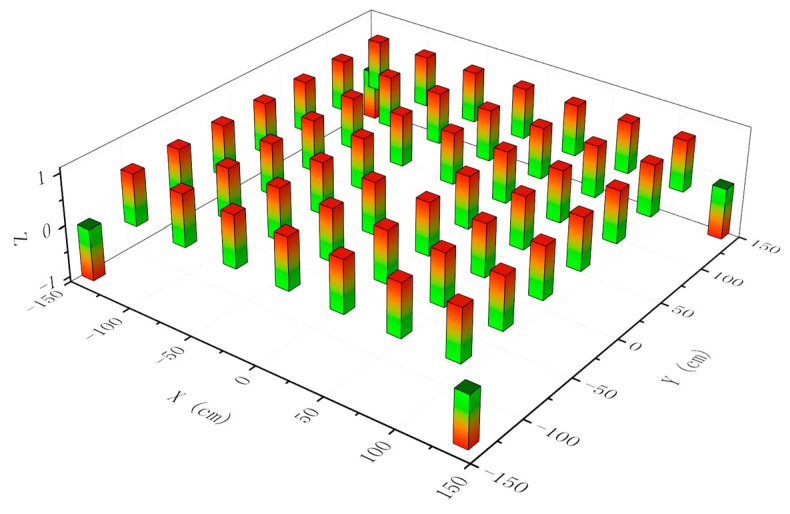
Symmetrical judgement in different positions. The X axis and Y axis represent the real square area, the unit is cm, and the center position is the coordinate origin of the X‒Y plane coordinate system. There are two possible values for Z: 1 means a correct judgement in this position while −1 means false.

Taking the center point as the reference, we divided the square into several small square grids according to the standard of seven rows and seven columns. The distance between each row or column was 40 cm; except for the center point, all grid intersection points were selected to test the performance of symmetrical judgement. In addition, four positions near the four corners were also selected. At these points of intersection, the robot carried a laser sensor running ROS system with SLAM and a symmetrical judgement program for different times. The program running results of symmetrical judgement are shown in Figure 12, except for the four corner positions; our method performed well and got almost all correct results. The reason why the robot fails to correctly judge a square environment in the four corner positions is explained in the simulation environment section—the laser sensor is too close to a wall or a corner. 

After a successful symmetrical judgement test, the robot needed to move toward the geometry center position and capture images for orientation decisions. We set up two circular areas with a radius of 5 mm or 10 mm; both shared the same center, which was also the center of the square environment. We counted the number of times that the mobile robot chassis moved to the circular areas. The statistical data are shown in Table 2; every time, the robot can reach the circular area where the radius length equals 15 mm. This error range is acceptable due to its small influence on the visual orientation decision. When the radius length equals 10 mm, the proportion is 94%. Even though the radius is 5 mm, the robot can still reach the smaller area 86% of the time. Each time the robot is placed around the geometric center in a different position; as a result, the deviation distance value and direction are also different. Thus, the final position error of the robot is evenly distributed in a circular area.

(2) Visual Features Extraction, Matching, and Making Decisions on Orientation

**Figure 13:**
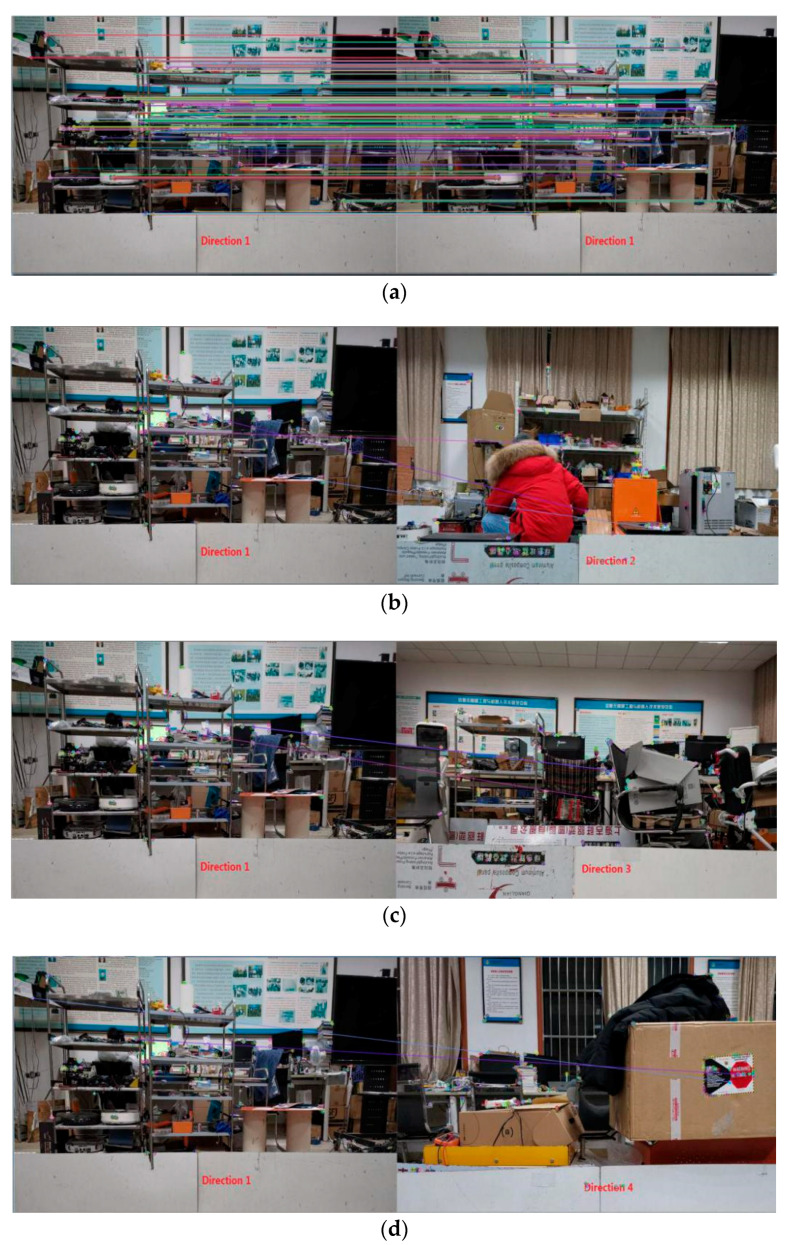
ORB features and matching lines. (**a**) Image captured from direction 1, doubled; (**b**) Image captured from direction 1 with that from direction 2; (**c**) Image captured from direction 1 with that from direction 3; (**d**) Image captured from direction 1 with that from direction 4.

We captured images from the four directions at the geometric center of the square environment, extracted ORB features, and tested the matching effect of the image pairs shown in Figure 13.

In Figure 13, the image captured from direction 1 was matched with all four images separately. We set a maximum value of 500, which was the number of ORB feature points. Figure 13a shows that almost all points from the same two images are matched successfully, and matched line pairs are parallel. Figure 13b shows three line pairs between images from direction 1 and direction 2. The number of line pairs can be ignored because it is too small; even so, the matches are in fact wrong. The same thing happens in Figure 13c,d, which both have two wrong matching lines. Similarly, matching the remaining three images with all four images will produce the same results. Therefore, we can be sure that it is feasible to use visual features as the basis of robot orientation judgment. 

However, features like ORB, in the case of a large number of images, are not robust and the image matching stage is time-consuming. We need to discretize a binary descriptor space, generate a more compact vocabulary, and convert each image into a visual word represented by a vector. The similarity results between different vectors are shown in Table 3.

The results in Table 3 lead us to the same conclusions as with ORB features matching. We find that the similarity value between images from different directions is small and close to 0, while that from the same direction is large and close to 1. The reason why the similarity between the same directions does not equal 1 is position error. A robot cannot move to an absolutely accurate position and keep the same orientation in different experiments.

**Figure 14:**
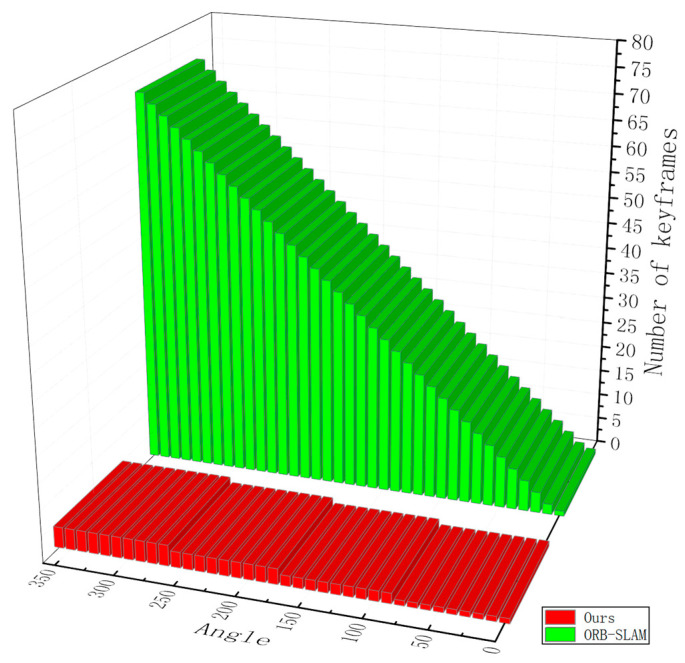
The number of keyframes achieved and maintained by the camera on a robot after rotating one circle in place. Our method needs just four images, while ORB-SLAM adds a new image for every 5° of rotation.

Our method, which uses sparse images to assist a laser slam system for localization, especially in the orientation decision part, has the advantage of less memory and disk storage use. We compared our method with ORB-SLAM, in which the number of images increases with the change of the robot rotation angle, as shown in Figure 14. Our method adds an image every 90°, while ORB-SLAM adds a new image for almost every 5° of rotation.

## 6. Discussion and Conclusions

This paper proposed a visual features-assisted method for robot localization in a symmetrical environment. The robot mainly used the laser SLAM system in ROS to map the surroundings in asymmetrical locations using the AMCL method. First, we proposed an approach to judge whether a closed environment was symmetrical, especially a square environment, as in the example given in this paper. Then, the robot moved to the geometric center position according to the results of laser scan data processing. Finally, the robot captured images, extracted features and converted them to a bag-of-words vector, and compared them to the visual map built before. When the orientation was determined, the rest of the localization work was easy to perform by the AMCL method using laser scan data and odometer information.

When a robot’s initial position is too close to one side of the walls or corners, the laser scans data are rough compared with the data achieved from the position near the center of an enclosed area. As a laser sensor has the same angular spacing between adjacent emission lines, the distance between two reflection points far from a laser sensor is large, while the distance between two reflection points closer to a laser sensor is small. These factors lead to misjudgments of the environment shape; fortunately, this problem can be solved by moving the robot away from the wall or corner. We can design an algorithm/program that allows the robot to analyze laser scan data away from walls or corners. In order to ensure accuracy and reliability, combined results from several laser scan data analyses will be adopted.

Although the method proposed in this paper can obtain effective localization results, the scene needs rich textural information to be extracted as features. When a robot is inside an empty, symmetrical room with a closed door and windows, with less textural information on the wall, our approach may fail. Therefore, our future research work will focus on line features or visual semantic information-assisted localization for an autonomous mobile robot in a symmetrical environment.

## Figures and Tables

**Table 1:** Correct recognition number of times in different symmetrical polygons (number of experiments in each environment = 50).

Polygon Environment	G‒V Distance (3 m)	G‒V Distance (5 m)	G‒V Distance (7 m)
Equilateral triangle	50	50	48
Square	50	50	50
Regular pentagon	50	50	50
Regular hexagon	50	50	49
Round	50	50	50

**Table 2:** Proportion of robot’s position in different areas.

	Radius Length of Circular Area or Error Range (mm)		
	R = 5	R = 10	R = 15
**Proportion (%)**	86	94	100

**Table 3:** Similarity between different images using a bag-of-words vector.

	Image 1	Image 2	Image 3	Image 4
Image 1	0.96	0.12	0.08	0.10
Image 2	0.12	0.93	0.07	0.08
Image 3	0.08	0.07	0.91	0.13
Image 4	0.10	0.08	0.13	0.95

## Data Availability

Not applicable.
